# Is the Evaluation of Quality of Life in NSCLC Trials Important? Are the Results to be Trusted?

**DOI:** 10.3389/fonc.2014.00173

**Published:** 2014-07-03

**Authors:** Vera Hirsh

**Affiliations:** ^1^Department of Medical Oncology, McGill University Health Centre, Royal Victoria Hospital, Montreal, QC, Canada

**Keywords:** HR QOL, symptoms, data collection, metastatic NSCLC, clinical trials

## Abstract

The majority of patients with non-small cell lung cancer present at the time of diagnosis with stage IV metastatic disease and they experience 2 or more disease-related symptoms. These symptoms may have a negative impact on their health-related quality of life (HR QOL). Data has shown many of these patients prefer a therapy to improve their symptoms rather than receive a therapy which slightly prolongs their survival without improving their symptoms. The improvement of disease-related symptoms on a specific drug or regimen augments the significance of prolongation of the progression-free survival or the response rate as well as symptom worsening. The choice of the questionnaires to evaluate patients’ reported outcomes and HR QOL benefits and the methods of collecting the data and their interpretations are very important. Only if the data are collected and analyzed properly will they be meaningful and can then be viewed as components that add the total value to a treatment and provide a comprehensive picture of the benefits and risks of a certain anticancer therapy.

## Introduction

Lung cancer is the leading cause of cancer death worldwide for both men and women (1). A majority of these patients present at the time of diagnosis with metastatic disease. Approximately 90% of patients with advanced non-small cell lung cancer (NSCLC) experience two or more disease-related symptoms such as cough, dyspnea, pain, anorexia, or fatigue (2). These symptoms in turn can cause psychological distress and may have a negative impact on a patient’s health-related quality of life (HR QOL). High degrees of psychological distress influence the emotional well-being in both patients and their families. In one survey, 68% of patients preferred a therapy that would improve disease-related symptoms without prolonging their life as opposed to treatment(s) that slightly prolonged their survival without improving symptoms (3).

A patient’s well-being can be affected both through symptom-control, treatment-related toxicity, and treatment efficacy. Therefore, treatments which can decrease the tumor burden and growth, and be less toxic, are very important for patients with advanced NSCLC (4, 5). It is of the utmost importance for these patients to preserve their independence and not be dependent on their loved ones feeling like a burden at the end of their lives (6–8).

Some studies suggest a link between tumor response and improvement of symptoms such as cough, dyspnea, chest pain, and also systemic symptoms such as fever, anorexia, and weight loss (9–11). The improvements in symptoms further augment the significance of good response rates or prolonged PFS. As the median OS of most of the patients with metastatic NSCLC is modest (around 1 year), with specific new targeted agents it approaches 2 years, therefore HR QOL and patients’ reported outcomes (PROs) carry high importance and thus will be reviewed here.

## Collection of the Data

Patients’ reported outcomes and HR QOL benefits are usually assessed during clinical trials using the self-administered cancer-specific European Organization for Research and Treatment of Cancer (EORTC) questionnaires QLQ C30 (12), the lung cancer-specific EORTC QLQ LC13 (13), and the Euro QOL EQ-5D (14) questionnaire (in afatinib LUX LUNG phase 3 trials or crizotinib phase 3 trials) or functional assessment of cancer therapy-lung (FACT-L) (15) (functional assessment of cancer treatment in lung cancer) questionnaire (i.e., in IPASS phase 3 trial with gefitinib). The QLQ C30 questionnaire consists of five functional scales (physical, role, cognitive, emotional, and social functioning), three symptom scales (fatigue, pain, and nausea/vomiting), a global health status/QOL scale, and single items, i.e., dyspnea, loss of appetite, constipation, diarrhea, sleep disturbance, and financial impact. The QLQ LC13 questionnaire incorporates one multi-item scale to assess dyspnea and a series of single items assessing cough, pain, sore mouth, dysphagia, peripheral neuropathy, alopecia, and use of pain medication.

For each scale/item, a linear transformation is applied to standardize the raw score on a range from 0 to 100 with 100 representing the best possible function/QOL for functional scales, and the highest burden of symptoms for symptom scales and symptom items. A 10-point change in an item or domain is perceived to be clinically meaningful (16). The percentage of patients who are classified as improved (≥10-point increase for functioning scales and ≥10-point reduction for symptom domains or items from baseline scores) with respect to each of the questionnaires is examined (16). In addition, the time-to-deterioration of an item/domain score is defined as the item from randomization to the first appearance of a score that is 10 points or more lower or higher than the baseline score (≥10-point reduction for functioning scales and ≥10-point increase for symptom scales or items).

The EQ-5D is a disease-generic questionnaire that comprises the EQ-5D and EQ-visual analog scale (VAS). The EQ-5D measures five dimensions of health (mobility, self-care, usual activities, pain/discomfort, and anxiety/depression). Each dimension comprises three levels (no problems, some/moderate problems, and extreme problems). Utility scores range from 0 to 1 and are calculated from the five EQ-5D items scores using the United Kingdom preference weights (17). The EQ-VAS records the patient’s self-rated health status on a vertical, graduated (0–100) VAS.

Functional assessment of cancer therapy-lung questionnaire (version 4) comprises 36 items across 5 domains/categories: physical, social, family, emotional, and functional well-being. The Lung cancer subscale consists of symptoms, cognitive function, and regret of smoking. Scores range from 0 (not at all) to 4 (very much) (15).

Each protocol specifies a schedule for questionnaires to be completed (at baseline, every 2–4 weeks, at the end of the treatment visit, and during the first follow-up visit). The use of concomitant medications is assessed at the baseline and during the trial, especially analgesic use, anti-anxiety/depression medications, O_2_ use, etc.

## Interpretation of the Results

Patients must answer the questionnaires prior to learning the results of their tests (scans) from their physicians in order to obtain reliable results. Help with the questionnaires should be available by knowledgeable staff in the clinic or hospital. Since patients must fill out the questionnaire by themselves, supervision of this procedure in order to ensure objectivity is important. Attention should be paid to the baseline scores. In randomized trials, are they well-balanced? Are they low (i.e., low burden of symptoms) or high (i.e., high burden of symptoms)? If the baseline scores are low, the percentage of patients with improved symptoms on certain anticancer treatments might be difficult to find. Delay of the symptom deterioration is usually of high importance. The longitudinal analysis which looks at symptoms and HR QOL over time (at different visit intervals) might be informative.

The compliance of patients a propos to the completion of their questionnaires must be reported at the baseline and also during the study. The compliance during the study should remain at ≥80% in order to interpret the results appropriately. In the case of EORTC questionnaires, both EORTC QLQ LC13 and QLQ C30 have to be analyzed to get a complete picture *not* only of lung cancer-related symptoms, but also of symptoms related to cancer treatment toxicities. The patients’ symptoms are treated by analgesics, cough suppressants, O_2_, anti-depressants, appetite stimulating agents, etc., and they all have to be incorporated in the final analysis. Other factors such as patient’s performance status (improving or deteriorating), weight loss, and special emotional counseling are of great value and can influence patients’ HR QOL.

## Conclusion

Patients’ reported outcomes and health-related quality of life outcomes are important parameters of the evaluation of new drugs or regimens of patients in advanced NSCLC, but only if the data are collected and analyzed correctly. They should be viewed as components of the total value of a treatment. They should provide, together with the other primary and secondary endpoints, a comprehensive picture of the benefits and risks of anticancer therapies for patients with metastatic NSCLC. This is the position taken by the Food and Drug Administration (18) and the European Medicine Agency (19, 20).

Dedicated personnel are required for this time-consuming process of collecting and analyzing the PROs and HR QOL data. The delivery of reliable results from these questionnaires requires the team work of knowledgeable and devoted workers. Consequently enabling patients with advanced NSCLC to feel more comfortable and independent during the last months or years of their life becomes a very important task in their treatment.

## Conflict of Interest Statement

The author declares that the research was conducted in the absence of any commercial or financial relationships that could be construed as a potential conflict of interest.
